# Nonpneumococcal Strains Recently Recovered from Carriage Specimens and Expressing Capsular Serotypes Highly Related or Identical to Pneumococcal Serotypes 2, 4, 9A, 13, and 23A

**DOI:** 10.1128/mBio.01037-21

**Published:** 2021-05-18

**Authors:** Robert E. Gertz, Fabiana C. Pimenta, Sopio Chochua, Shanda Larson, Anne-Kathryn Venero, Godfrey Bigogo, Jennifer Milucky, Maria da Gloria Carvalho, Bernard Beall

**Affiliations:** a Respiratory Diseases Branch, Division of Bacterial Diseases, National Center for Immunization and Respiratory Diseases, Centers for Disease Control and Prevention, Atlanta, Georgia, USA; b IHRC Incorporated, Atlanta, Georgia, USA; c Kenya Medical Research Institute, Centre for Global Health Research, Kisumu, Kenya; University of Mississippi Medical Center

**Keywords:** capsular polysaccharide, capsular serotypes, commensal species, pneumococcal

## Abstract

The polysaccharide capsule is a key virulence factor of Streptococcus pneumoniae. There are numerous epidemiologically important pneumococcal capsular serotypes, and recent findings have demonstrated that several of them are commonly found among nonpathogenic commensal species. Here, we describe 9 nonpneumococcal strains carrying close homologs of pneumococcal capsular biosynthetic (*cps*) loci that were discovered during recent pneumococcal carriage studies of adults in the United States and Kenya. Two distinct Streptococcus infantis strains cross-reactive with pneumococcal serotype 4 and carrying *cps4*-like capsular biosynthetic (*cps*) loci were recovered. Opsonophagocytic killing assays employing rabbit antisera raised against *S. infantis* US67cps4 revealed serotype 4-specific killing of both pneumococcal and nonpneumococcal strains. An *S. infantis* strain and two Streptococcus oralis strains, all carrying *cps9A-*like loci, were cross-reactive with pneumococcal serogroup 9 strains in immunodiffusion assays. Antiserum raised against *S. infantis* US64cps9A specifically promoted killing of serotype 9A and 9V pneumococcal strains as well as S. oralis serotype 9A strains. Serotype-specific PCR of oropharyngeal specimens from a recent adult carriage study in the United States indicated that such nonpneumococcal strains were much more common in this population than serotype 4 and serogroup 9 pneumococci. We also describe S. oralis and *S. infantis* strains expressing serotypes identical or highly related to serotypes 2, 13, and 23A. This study has expanded the known overlap of pneumococcal capsular serotypes with related commensal species. The frequent occurrence of nonpneumococcal strains in the upper respiratory tract that share vaccine and nonvaccine capsular serotypes with pneumococci could affect population immunity to circulating pneumococcal strains.

## INTRODUCTION

Streptococcus pneumoniae is an opportunistic pathogen restricted primarily to humans. Its principal virulence factor is its polysaccharide capsule, currently known to have at least 100 different serotypes (1). Ninety-eight of these capsular polysaccharides are synthesized via the so-called Wzx/Wzy-dependent pathway, dependent upon a specific repeat unit membrane transporter (Wzx), specific oligosaccharide polymerase (Wzy), and glycosidic linkage to peptidoglycan (2–5). Many serotypes are grouped into structurally related serogroups. For example, serogroup 9 consists of two pairs of highly related serotypes, 9A/9V and 9L/9N. Other capsular polysaccharides, such as types 1, 2, 4, and 5, are structurally and serologically distinct from other known capsules. The pneumococcal capsular biosynthetic (*cps*) loci form 8 clusters based upon gene homology groups and sequence similarities (3).

Of the 5 serotypes that were first identified in pneumococci (6–8), 3 of them (serotypes 1, 2, and 5) have recently been characterized from nonpneumococcal commensal species (9–12). Further, in recent adult upper respiratory tract (URT) carriage assays carried out in the United States and Kenya, nonpneumococcal species expressing these and several other serotypes appear to be more common than pneumococci of the same serotypes (9, 10, 13–15). The observed directionality of horizontal transfer events, where pneumococci are most often observed as recipients of related mitis group DNA (16), and the abundance of *cps* loci shared between pneumococci and its close relative commensal species that reside in the URT in higher density (9, 10, 13), is consistent with pneumococcal serotype diversity originating in non-pneumococcal species. That said, species origins for shared serotypes, such as serotypes 1 and 5, have not been formally demonstrated (9, 10).

Here, we describe strains of S. oralis and *S. infantis* from adult carriage expressing counterparts of serotypes commonly expressed by important disease-causing strains of pneumococci, including PCV serotypes 4 and 9V, the 23-valent purified polysaccharide vaccine serotype 2, and nonvaccine serotypes 23A and 13. To our knowledge, with the exception of serotype 2 (11, 12), these serotypes have not been previously observed in nonpneumococcal species, and it is not known how common such strains are in their natural reservoir in the human URT.

## RESULTS

### Nonpneumococcal strains PCR positive for pneumococcal serotypes 4, 9A/V, 18A/B/C/F, and 23A recovered from U.S. adult carriage study.

In a recent study of paired nasopharyngeal/oropharyngeal (NP/OP) specimens from 460 adults, 450 individuals (908 of 920 specimens) were culture negative for pneumococci (10). Isolation and characterization of nonpneumococcal strains of PCV serotypes 1 and 5 associated with the pneumococcal surveillance project (15) have been described (9, 10), as have nonpneumococcal counterparts of *cps12F* and *cps33F* (9).

Despite comprehensive attempts to recover pneumococci from all 460 paired specimens (10), there were no pneumococci recovered of serotype 4 or of serogroups 9 and 18. Only 10 individuals yielded pneumococcal isolates, including 3 that were serotype 23A (10). Fifty (47 OP and 3 NP) specimens from the 450 individuals negative for pneumococcal culture were PCR positive for serotype 4 (PCR4^+^) (Table 1). Two independent PCR4^+^ OP specimens (of the eight screened) yielded PCR4^+^, alpha-hemolytic colonies that were not pneumococci, since they were bile insoluble and negative for the CDC pneumococcal *lytA* quantitative PCR (qPCR) assay (17) (strains US302cps4 and US67cps4). Two nonpneumococcal PCV9A/9V^+^ OP isolates were recovered from this carriage survey (strains US64cps9A and US3cps9A) from two different OP specimens among the 41 total PCR9A/V^+^ specimens (Table 1). Numerous specimens were also PCR serogroup 18 positive (PCR18^+^ or PCR23A^+^), and, subsequently, single nonpneumococcal PCR18^+^ and PCR23A^+^ strains were recovered (US164cps18 and US1133cps23A, respectively). All 6 nonpneumococcal strains (optochin resistant and bile insoluble) were recovered from OP specimens (Table 1). Of the 136 specimens PCR positive for serotypes 4, 9A/V, 23A, and 18A/B/C/F, 130 were OP specimens, consistent with the oropharynx providing the primary reservoir for nonpneumococcal strains that carry homologs of pneumococcal *cps* loci (13). In contrast, NP specimens are relatively enriched for pneumococcal strains (18).

**TABLE 1:** Summary of PCR serotype and culture-positive results for serotypes 4, 9A/9V, 18C, and 23A from paired NP and OP specimens^*c*^

PCR serotype	No. of individuals PCR positive for indicated serotype (specimens)	No. of swabs screened for nonpneumococcal strains positive for indicated PCR-serotype^*a*^/no. of colonies screened/no. of culture-positive swabs for non-Spn strain of indicated PCR serotype	PCR serotype-positive strain(s) isolated (species)^*a*^^,^^*b*^
4	50 (47 OP, 3 NP)	8/122/2	US302cps4 and US67cps4 (both *S. infantis*)
9A/9V	41 (all OP)	3/50/2	US3cps9A (S. oralis), US64cps9A (*S. infantis*)
18A/B/C/F	36 (all OP)	2/68/1	US164cps18 (*S. infantis*)
23A	9 (6 OP, 3 NP)	1/84/1	US1133ps23A (*S. infantis*)
Total	136 (130 OP, 6 NP)		

a Attempts were made to isolate pneumococci from all specimens (both negative and positive for the CDC pneumococcal qPCR *lytA* assay).

b All nonpneumococcal strains were negative for the CDC pneumococcal qPCR *lytA* assay and were recovered from OP specimens.

c Specimens were taken from 450 pneumococcal culture-negative individuals.

### PCR4^+^ nonpneumococcal strains have capsules serologically cross-reactive with serotype 4.

**FIG 1:**
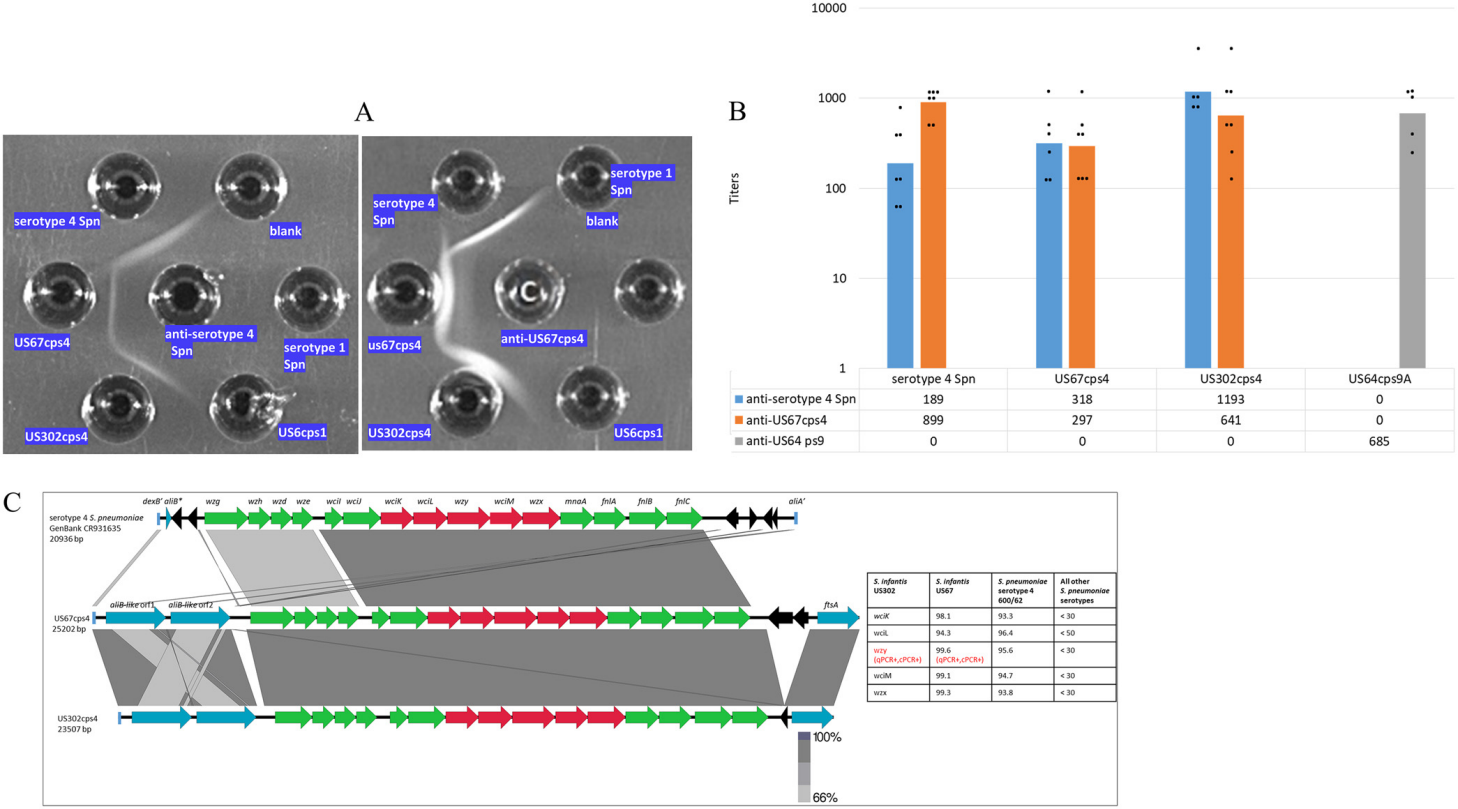
(A) Reactivity of pneumococcal (Spn) typing serum (left) and antiserum raised against US67cps4 (right) against serotype 4 pneumococcal strain and two nonpneumococcal strains carrying *cps4-*like operons (US67cps4 and US302cps4). Negative-control strains include a serotype 1 pneumococcal strain and serotype 1 Streptococcus mitis strain US6cps1 (10). (B) Cross-species and serotype 4-specific opsonophagocytic killing activity of antisera prepared against serotype 4 S. pneumoniae and nonpneumococcal strain US67cps4. Geometric mean titer (GMT) values are shown with >50% killing compared with growth in the complement control wells. Titers used to calculate GMTs are indicated with black circles. (C) Alignment of *cps4* loci from serotype 4 pneumococci and nonpneumococcal strains US6cps4 and US302cps4 recovered from adult OP specimens. The pneumococcal sequence between the 3′ end of *dexB* (*dexB*′) and the 5′ end of *aliA* (*aliA*′) is aligned with corresponding sequences from the two nonpneumococcal strains that lie downstream of *dexB.* The *cps4* operon genes indicated in green are relatively highly conserved with counterparts from *cps* operons other than *cps4*, while genes in red indicate genes that share >94% sequence identity only within *cps4* counterparts (indicated in the accompanying table as well as indication of positivity of the 2 strains with qPCR and cPCR assays for serotype 4). Also indicated in the table is the tentative assignment of species *S. infantis* due to characteristic gene arrangement at this genomic region. Genes indicated in blue are unrelated to capsule biosynthesis. A remnant of an *aliB* gene is indicated (aliB*) in the pneumococcal strain. Remnants of transposase genes are indicated in black. Black lines are indicative of small localized homologous segments. The different gray shades shared between the *aliB-*like genes are indicative of high homology shared between all 4 genes.

Although weak compared to serotype 4 pneumococci, positive Quellung reactions were seen for nonpneumococcal strains US302cps4 and US67cps4 employing pneumococcal type 4 antiserum (not shown), which corresponded to specific positivity of US302cps4 and US67cps4 extracts in immunodiffusion experiments employing this same antiserum (Fig. 1A). Antiserum prepared against strain US67cps4 also strongly and specifically reacted with these same extracts (Fig. 1A). Nonpneumococcal and pneumococcal strains of various serotypes other than type 4 were unreactive in immunodiffusion assays employing these two antisera, as shown for serotype 1 S. mitis (10) and S. pneumoniae.

### Cross-species serotype 4-specific OPK.

There was reproducible opsonophagocytic killing (OPK) activity against nonpneumococcal strains US302cps4 and US67cps4 employing serotype 4-specific pneumococcal typing antiserum but none detected employing control antiserum against strain US64cps9A (Fig. 1B). Antiserum prepared against US67cps4 also exhibited serotype-specific OPK against serotype 4 pneumococci and the two nonpneumococcal PCR4^+^ strains, while antiserum against US64cps9A lacked OPK activity against US67cps4, US302cps4, and serotype 4 pneumococci.

### Nonpneumococcal *cps* loci.

The pneumococcal *cps4* locus is one of 7 cluster 1 *cps* loci (3) that shares features with *cps5* and *cps12* loci from nonpneumococcal species (9). Serotype 4 pneumococci contain *N*-acetyl-l-fucosamine in their capsules, corresponding to the synthetic genes *fnlA* to *fnlC* (*fnlA-fnlC*) (3, 5), which were also observed in the two nonpneumococcal counterparts (Fig. 1C). These two strains were tentatively assigned as *S. infantis* due to the characteristic downstream location of the cell division gene *ftsA* and upstream location of two *aliB*-like genes (9, 11). The *cps4* loci between the species also share highly related *wciI* (initial sugar transferase) and *wciJ* (glucosyl transferase) genes. The shared seroreactivity between serotype 4 pneumococci and these two nonpneumococcal strains, observed in Quellung reactions (not shown), immunodiffusion experiments (Fig. 1A), and OPK assays (Fig. 1B), is consistent with highly conserved serotype 4-specific genes.

Also consistent with shared ancestry and close structural relatedness between serotype 4 pneumococcal capsules and capsules from the two nonpneumococcal PCR4^+^ strains US67cps4 and US302cps4 was the gene order shared between the three 16,368- to 16,429-bp loci and very close relatedness (93.3 to 96.4% sequence identity) between alleles of the 5 central genes that encode serotype-specific synthetic functions (Fig. 1C). The 5 genes include the repeat unit oligosaccharide synthase gene (*wzy*), which shared ∼96% sequence identity between species and allowed PCR detection employing conventional PCR (cPCR) (19) and qPCRs (20) specific for serotype 4.

### PCR9A/9V^+^ nonpneumococcal strains have capsules serologically related to serogroup 9 and specifically react with typing factor serum 9d.

Three PCR9AV^+^ nonpneumococcal specimens and strains retrospectively isolated from pneumococcal carriage specimens (10, 14) were positive for the qPCR-serotyping reaction targeting *wzx9A-wzx9V* (20). One of the three PCR9AV^+^ strains (US64cps9A) was also positive for the cPCR assay targeting *wzx9A-wzx9V* (21). Positive Quellung reactions were observed for the three nonpneumococcal PCR9AV^+^ strains when employing pooled serogroup 9 antisera containing all 4 factors for resolution of serotypes 9A, 9V, 9L, and 9N pneumococci (not shown) but not when the 3 strains were reacted with 3 of the individual factors used for resolving serogroup 9 (factors 9e, 9g, and 9f). US64cps9A displayed an obviously stronger Quellung result, with pooled serogroup 9 antisera compared to the other two PCR9AV^+^ strains, that was slightly more noticeable than the reactivity with the serotype 9A pneumococcal reference strain. Barely detectable positive Quellung reactions were observed for the 3 PCR9AV^+^ nonpneumococcal strains when reacted with factor 9d (reacts with pneumococcal serotypes 9A and 9V only). According to this result, these 3 strains are tentatively serotype 9A, since they did not quell with the 9V-specific factor 9g. Strains of all four pneumococcal serogroup 9 serotypes, and all 3 PCR9AV^+^ nonpneumococcal strains, displayed strongly positive Quellung reactions when mixed with antiserum prepared against US64cps9A (data not shown). Since serotype 9A capsule contains glucuronic acid, these strains were subjected to an assay measuring uronic acids (22–24). All three nonpneumococcal PCR9AV^+^ strains yielded uronic acid assay values corresponding to more than 100 μg/ml, as predicted by the glucuronic acid standard, with strain US64cps9A giving higher readings than both pneumococcal and nonpneumococcal serotype 9A strains (see Fig. S1 in the supplemental material).

10.1128/mBio.01037-21.1 FIG S1 Left chart depicts uronic acid assays comparing capsule amounts from pneumococcal reference serotype 2 strain compared to S. oralis strain KE9746cps2 and the reference pneumococcal serotype 9A strain compared with two *S. infantis* strains (KE9676cps9A and US64cps9A) and one S. oralis strain (US3cps9A). The capsular-negative pneumococcal strain R6 and S. mitis strain 50014 were used as negative controls. Two independent cultures were assessed for each strain, with 6 to 8 readings for each. The average values are shown, with value ranges depicted by vertical lines. The right chart depicts absorbance for concentrations of glucuronic acid ranging from 5 to 1,000 μg/ml. Aliquots (200 μl) of each indicated concentration were added to 12.5 mM sodium tetraborate in concentrated sulfuric acid, mixed well, heated to 100°C, transferred to an ice bath, had 20 μl 0.15% m-hydrobiphenyl in 0.5% NaOH added, and were transferred to borosilicate tubes for readings at 520 nm. Download FIG S1, PDF file, 0.06 MB. Copyright © 2021 Gertz et al. 2021 Gertz et al. https://creativecommons.org/licenses/by/4.0/ This content is distributed under the terms of the Creative Commons Attribution 4.0 International license.

**FIG 2:**
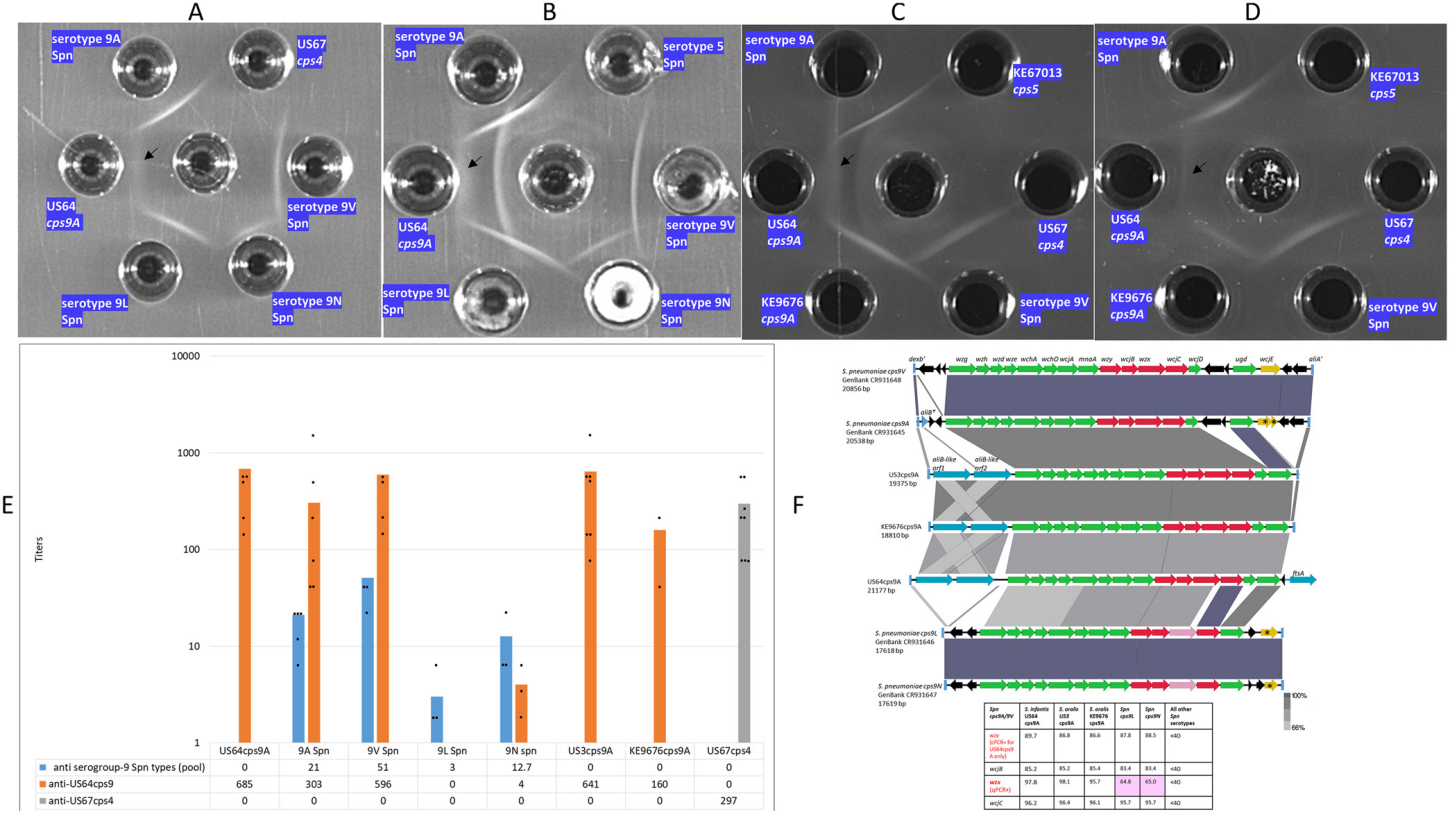
(A) Cross-reactivity of nonpneumococcal strains with pneumococcal typing sera against serogroup 9. Pooled pneumococcal serogroup 9 typing factors (center) are reacted against extracts of pneumococcal serotypes 9A, 9L, 9N, and 9V. A negative-control extract of nonpneumococcal strain US67cps4 is included. A faint serogroup 9 precipitate (arrow) is visible between US64cps9A extract and the centrally placed pooled pneumococcal serogroup 9 typing antisera. (B) Anti-US64cps9A sera (center) reacted against extracts of pneumococcal serotypes 9A, 9L, 9N, 9V, and US64cps9A. A negative-control extract of control serotype 5 S. pneumoniae is included. The antiserum-distal precipitate corresponds to the precipitate showing identity with the corresponding precipitate from KE9676cps9A in panel C. (C) Anti-US64cps9A sera (center) reacted against extracts of serotype 9A Spn, US64cps9A, KE9676cps9A, and serotype 9V Spn. Negative controls of nonpneumococcal strain US67cps4 and KE67013cps5 are included. The faint precipitate from US64cps9A (arrow) shows apparent serologic identity to the corresponding precipitate from KE9676cps9A. (D) Pooled serogroup 9 typing factors (center) reacted against extracts of serotype 9A Spn, US64cps9A, KE9676cps9A, and serotype 9V Spn. Negative-control extracts of nonpneumococcal strains US67cps4 and KE67013cps5 are included. A faint serogroup 9 precipitate (arrow) is visible between US64cps9A extract and the centrally placed pooled Spn serogroup 9 typing antisera and appears to share serologic identity with the corresponding precipitate from KE9676cps9A. (E) Cross-species and serotype 9A/9V-specific opsonophagocytic killing activity of antisera prepared against serotype 9A nonpneumococcal strain US64cps9A and pooled antisera against S. pneumoniae serogroup 9 serotypes. Geometric mean titer (GMT) values are shown with >50% killing compared with growth in the complement control wells. Titers used to calculate GMTs are indicated with black circles. (F) Alignment of *cps* loci from serogroup 9 pneumococci and nonpneumococcal strains US3cps9A, US64cps9A, and KE9676cps9a recovered from adult OP specimens. The pneumococcal sequence between the 3′ end of *dexB* (*dexb*′) and the 5′ end of *aliA* (*aliA*′) is aligned with corresponding sequences from the three nonpneumococcal strains that lie downstream of *dexB.* The *cps* operon genes indicated in green are relatively highly conserved with counterparts from *cps* operons other than from serogroup 9, while genes in red share >83% sequence identity among the 4 pneumococcal serogroup 9 operons, with the exception of *wzx*, which distinguishes 9A/9V from 9L/9N (indicated in pink and in the accompanying table), and indicates positivity of the 3 nonpneumococcal strains with *wzx-*specific qPCR. The active *wcjE* acetylase gene in *cps9V* is indicated in gold, and inactive forms in the other 3 pneumococcal types are indicated with an asterisk. The nonpneumococcal *cps9A-*like operons lack *wcjE* genes. Genes indicated in blue are not involved in capsule biosynthesis. A remnant of an *aliB* gene is indicated (aliB*) in one pneumococcal strain. Remnants of transposase genes are indicated in black. The different gray shades shared between the *aliB-*like genes are indicative of the high homology shared between all 4 genes.

Immunodiffusion assay results employing serogroup 9 typing sera and anti-US64cps9A (Fig. 2A to D) were in agreement with Quellung testing. A faint precipitate is shown for US64cps9A extract reacted with pooled serogroup 9 sera in Fig. 2A, with clear precipitate bands shown for each of the 4 serogroup 9 pneumococcal strains. Despite positive factor 9d Quellung results with serotypes 9A and 9V pneumococci as well as the three nonpneumococcal PCR9A/V^+^ strains (not shown), reproducible immunoprecipitates were not apparent when reacting factor 9d with any pneumococcal or nonpneumococcal serogroup 9 strains (data not shown).

Antiserum prepared against US64cps9A reacted with each of the 4 serogroup 9 pneumococcal serotypes (Fig. 2B and C). This antiserum produced a diffuse immunoprecipitate close to the US64cps9A extract (compare to same extract reacting with pooled serogroup 9 antiserum in Fig. 2A) and a sharper band close to the central antiserum well (Fig. 2C). Neither of these two bands was evident by employing various extracts from PCR9AV-negative nonpneumococcal strains. Both US3cps9A (not shown) and KE9676cps9A showed the same reactivity to these antisera as US64cps9A (Fig. 2C and D), with the exception of the absence of the apparently US64cps9A-specific precipitate (antiserum-proximal) shown in Fig. 2B and, to a lesser extent, in Fig. 2C. Presumably this relatively strong precipitate line is due to a distinct surface antigen unrelated to the 9A polysaccharide that is expressed by US64cps9A and not expressed by the other strains shown.

### Cross species-specific opsonophagocytic activities against serotypes 9A and 9V.

Consistent with observed reactivity in Quellung reactions with pooled serogroup 9 pneumococcal typing antisera and with factor 9d, antiserum against US64cps9A was highly active in OPK assays against pneumococci of serotypes 9A and 9V with low OPK activity against pneumococci of serotypes 9L and 9N (Fig. 2E). Employing anti-US64cps9A, high OPK activity was observed against serotypes 9A and 9V pneumococci and against strains US3cps9A, US64cps9A, and KE9676cps9A. No OPK activity was observed against US67cps4. Pooled serogroup 9 pneumococcal typing antisera showed modest OPK activity against pneumococci of serotypes 9A, 9V, and 9N but low activity against serotype 9L pneumococci and no observable OPK activity against the three *cps9A*-positive nonpneumococcal strains. Antisera raised against serotype 4 strains had no activity against serotype 9A pneumococci or putative serotype 9A nonpneumococcal strains.

### US64cps9A, US3cps9A, and KE9676cps9A *cps* operons.

The four pneumococcal serogroup 9 capsular polysaccharide structures are very similar, and their *cps* loci comprise cluster 6 (3, 5). The 5-unit repeating structures of serotypes 9A/9V, 9N, and 9L are very similar but distinct from each other. The pneumococcal serotype 9A/9V *wzx* (flippase) gene is more discriminatory than *wzy* (repeat unit polymerase gene) when distinguishing between the two syntenic pneumococcal *cps* pairs (9A/9V and 9L/9N), and the pneumococcal *wzx* is much more homologous than *wzy* to their counterparts in the three nonpneumococcal PCR9/A^+^ strains (Fig. 2F). It follows that the *wzx9V-9A-*specific qPCR assay (19) was positive for these 3 strains while *wzy-*directed cPCR (21) was negative for 2 of the 3 strains (Fig. 2F).

Serotypes 9V and 9A share the same repeating oligosaccharide subunit structure that differs between them only by lack of WcjE-mediated O-acetylation in relatively rare serotype 9A strains that arise from mutation of the *cps9V* O-acetyltransferase gene, *wcjE* (3, 5, 25). The serotype 9A/9V-specific OPK activity (Fig. 2E), the specific Quellung positivity with serotyping factor 9d (not shown), the high interspecies similarity between the *cps9A*-*cps9V* flippase gene (*wzx*) alleles, the presence of the *wcjD* O-acetyltransferase gene (absent in serotypes 9L and 9N), and the lack of a *wcjE* homolog (Fig. 2F) are all features consistent with these three serogroup 9 nonpneumococcal strains expressing the serotype 9A polysaccharide.

Judging from previous observations (9, 11), the *dexB* and *ftsA* genes flanking the putative *cps9A* locus within US64cps9A together with the upstream two full-length *aliB*-like genes was suggestive of *S. infantis.* The *cps* region organization in strains US3cps9A and KE9676cps9A (flanking *dexB* and *aliA*, two full-length *aliB*-like genes) was suggestive of S. oralis (Fig. 2F).

### Cross-reactivity of US1133cps23A with pooled serogroup 23 sera and reactivity of anti-US1133cps23A with pneumococcal serogroup 23 serotypes.

**FIG 3:**
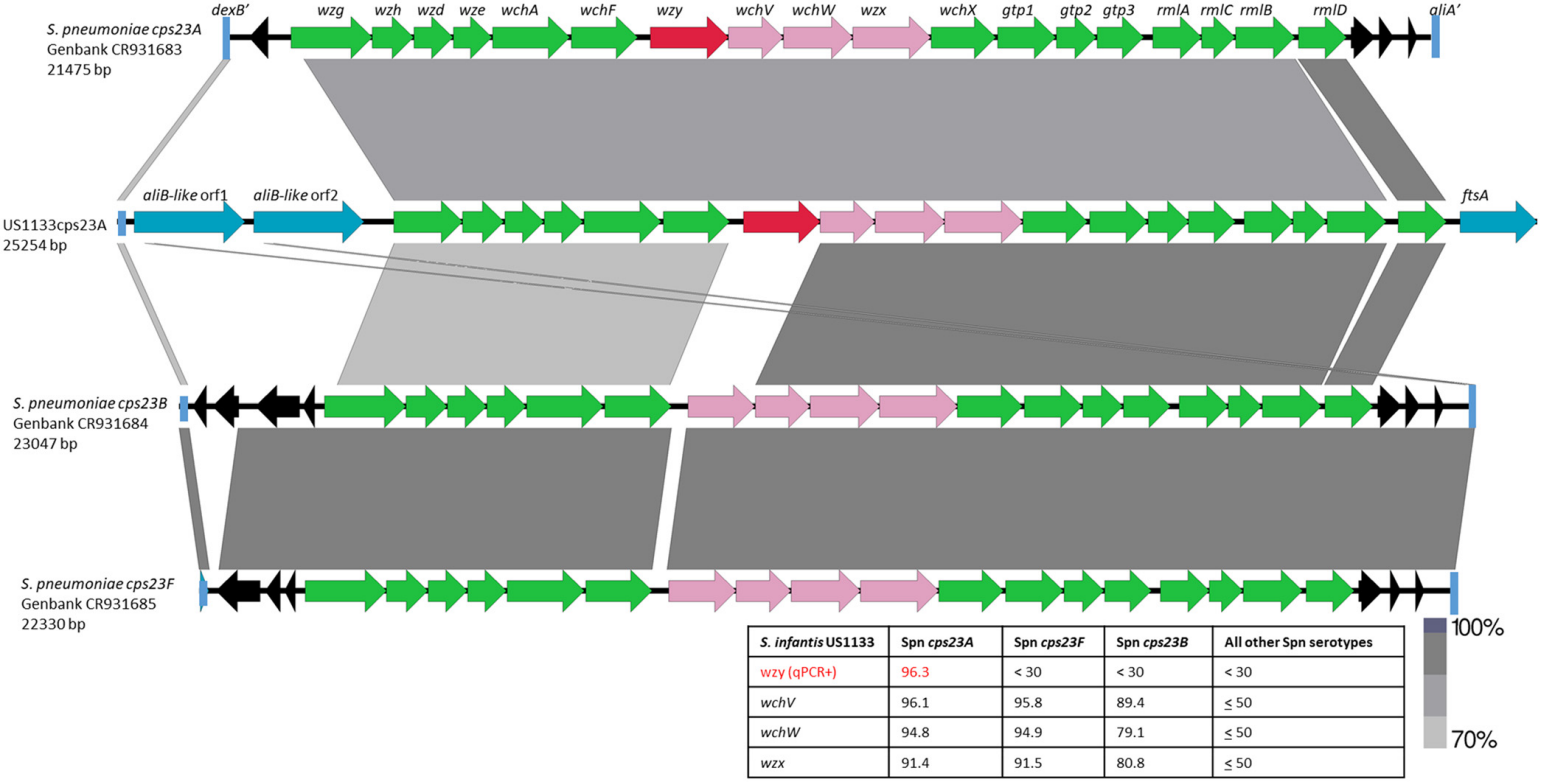
Alignment of *cps* loci from serogroup 23 pneumococci and nonpneumococcal strains US1133cps23A recovered from adult OP specimens. The pneumococcal sequence between the 3′ end of *dexB* (*dexB*′) and the 5′ end of *aliA* (*aliA*′) is aligned with corresponding sequence from US1133cps23. The *cps* operon genes indicated in green are relatively highly conserved with counterparts from non-serogroup 23 *cps* operons, while the *wzy* gene alleles in red depict dissimilarity to all other known pneumococcal *cps* genes. Genes indicated in pink share ≥83% sequence identity between 2 and 3 corresponding serogroup 23 *cps* genes. Genes indicated in blue are not involved in capsule biosynthesis. Remnants of transposase genes are indicated in black.

Nonpneumococcal strain US1133cps23A was recovered through screening for *wzy23A*-positive (PCR23A^+^) isolates from an OP specimen (Table 1) using qPCR (20). Pooled antiserum against the three serogroup 23 serotypes 23A, 23B, and 23F produced weak positive Quellung reactions with US1133cps23A (not shown); however, individual serogroup 23 factor sera did not produce detectable results. Antisera raised against nonpneumococcal strain US1133cps23A quelled pneumococcal strains of serotypes 23A, 23B, and, more weakly, 23F (not shown). The strongest Quellung reactivity of anti-US1133cps23A was observed with serotype 23A pneumococci. It is notable that antiserum prepared against serotype 23F pneumococci cross-reacts very little with serotype 23A and not at all with 23B, which correlates to their known polysaccharide structures (26, 27). The high sequence identity between the US1133cps23A and pneumococcal *cps23A wzy* alleles is consistent with the possibility of an identical capsular chemical structure shared between US1133cps23A and serotype 23A pneumococci (Fig. 3).

Despite the positive Quellung results employing pooled serogroup 23 pneumococcal typing serum and antiserum prepared against US1133cps23A, we did not see US1133cps23A extract reactivity in immunodiffusion experiments with pooled serogroup 23 typing antisera. Antisera raised against US1133cps23A did not visibly react with the three serogroup 23 pneumococcal serotypes and generated only a weak band against US1133cps23A extract (not shown).

### Nonpneumococcal serotype 2 Kenya carriage OP strain.

**FIG 4:**
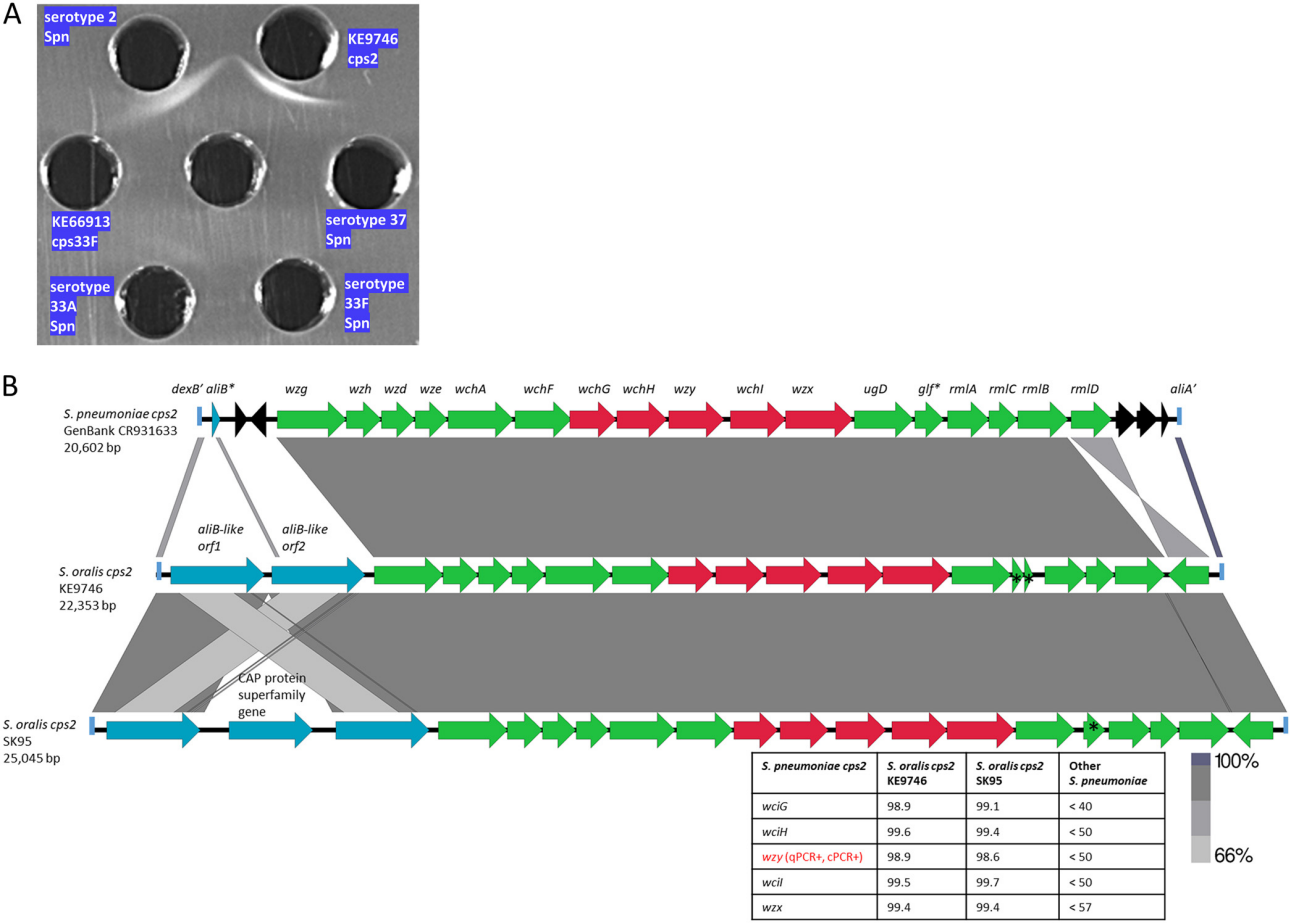
(A) Typing antiserum against pneumococcal serotype 2 reacted against serotype 2 S. pneumoniae and S. oralis strain KE9746cps2. A control S. oralis strain carrying a cps33F-like locus and pneumococcal serotypes 33A, 33F, and 37 are also included. (B) Alignment of *cps2* loci from serotype 2 pneumococcal reference and nonpneumococcal strains: KE9746cps2 recovered from adult OP specimens and previously described serotype 2 S. oralis strain SK95 (11, 12). The pneumococcal sequence between the 3′ end of *dexB* (*dexB*′) and the 5′ end of *aliA* (*aliA’*) is aligned with corresponding sequences from the two nonpneumococcal strains that lie downstream of *dexB.* The *cps2* operon genes indicated in green are relatively highly conserved with counterparts from *cps* operons other than *cps2*, while genes in red indicate genes that share >99% sequence identity only within *cps2* counterparts (indicated in the accompanying table as well as the indication of positivity the 2 strains with qPCR and cPCR assays for serotype 2). Genes indicated in blue are unrelated to capsule biosynthesis. A remnant of an *aliB* gene is indicated (aliB*) in the pneumococcal strain. Remnants of transposase genes are indicated in black. The different gray shades shared between the *aliB-*like genes are indicative of the high homology shared between all 4 genes. Truncated and presumably inactive *glf* gene segments (required for galactopyranose synthesis) are indicated with asterisks.

Due to the extreme abundance and allelic diversity of serotype 2-specific amplicons from apparent pneumococcus-negative URT specimens recovered during a pneumococcal carriage survey performed in Kenya during 2009 (13), we attempted to recover a nonpneumococcal PCR2^+^ strain from recent (2013) carriage specimens recovered from adults in the same area (14). Although there were no serotype 2 pneumococci recovered among 2,590 adults, a nonpneumococcal strain (KE9746cps2) that was *wzy2* positive by cPCR (21) and tested strongly Quellung positive for serotype 2 was recovered from an adult OP specimen. This result correlated with a pronounced serotype 2-specific immunoprecipitate in immunodiffusion (Fig. 4A) and higher uronic acid assay values than from the corresponding serotype 2 reference strain (Fig. S1). The KE9746cps2 *cps2* operon closely resembled the pneumococcal cluster 2 *cps2* operon (3) and *cps2* from recently described serotype 2 S. oralis strains (11, 12) (Fig. 4B).

### U.S. serotype 13 NP strain.

**FIG 5:**
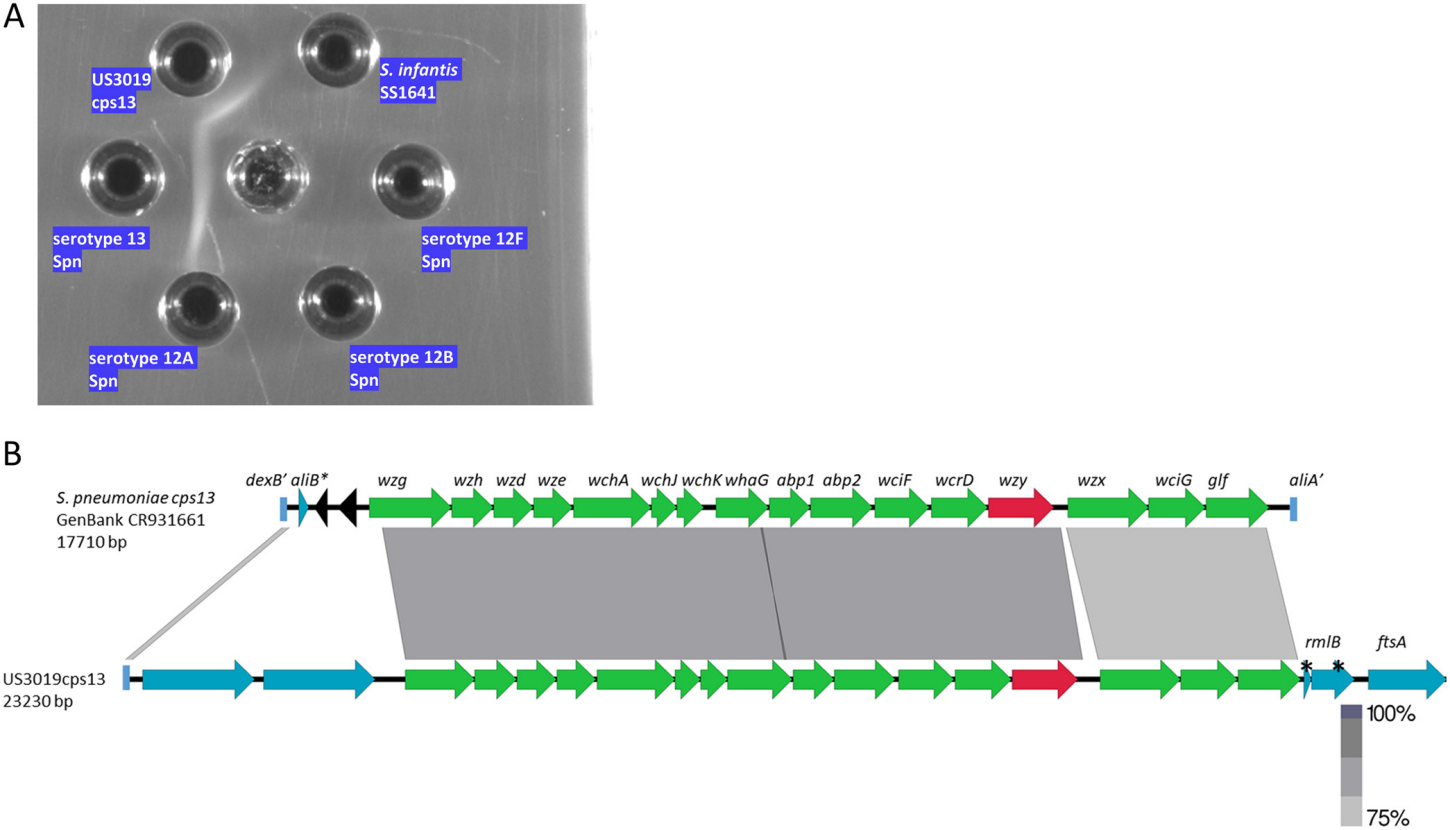
(A) Pneumococcal typing antiserum against serotype 13 (center well) reacted against extracts of nonpneumococcal strain US3019cps13 and the serotype 13 pneumococcal reference strain. Also included are negative-control extracts of *S. infantis* strain SS1641 and serogroup 12 pneumococcal extracts. (B) Alignment of *cps13* loci from the serotype 13 pneumococcal reference and nonpneumococcal strain US3019. The pneumococcal sequence between the 3′ end of *dexB* (*dexB*′) and the 5′ end of *aliA* (*aliA’*) is aligned with corresponding sequence from US3019cps13 that lies downstream of *dexB.* The *cps13* operon genes indicated in green are relatively highly conserved with counterparts from *cps* operons other than *cps13* (up to 87% identity), while *wzy13* shares >99% sequence identity only within *cps13* counterparts and shares 91% sequence identity with US3019cps13. Genes indicated in blue are not associated with capsule biosynthesis. A remnant of an *aliB* gene is indicated (aliB*) in the pneumococcal strain. Remnants of transposase genes are indicated in black. Inactive remnants of an *rmlB* rhamnose biosynthetic gene are indicated by asterisks.

US3019cps13 was fortuitously sent to the CDC Streptococcus laboratory from an adult prison inmate among one carriage isolate and three invasive isolates recovered during a serotype 12F invasive pneumococcal disease (IPD) outbreak and was the sole isolate in this study identified initially employing the Quellung reaction rather than through PCR serotyping. Among the 4 isolates sent to the CDC, the 3 invasive isolates were serotype 12F pneumococcal strains (bile soluble) and single NP isolate was serotype 13 and not bile soluble. Extracts of US3019cps13 provided serotype 13-specific immunoprecipitates when reacted against pneumococcal typing serum (Fig. 5A).

The *cps13* locus represents one of 23 different cluster 4 pneumococcal serotypes (3). The apparent serologic identity between US3019cps13 and serotype 13 pneumococci corresponded to the high homology (68 to 94% sequence identity) shared between each gene conserved of the 16 gene *cps* operons (Fig. 5B). The *wzy13* gene, which shares 91% sequence identity with its counterpart in US3019cps13, is the most divergent and unique of the pneumococcal *cps13* operon genes in that it shares <50% identity with all other known *cps* genes from other serotypes.

### US164cps18 nonpneumococcal strain from U.S. carriage survey.

**FIG 6:**
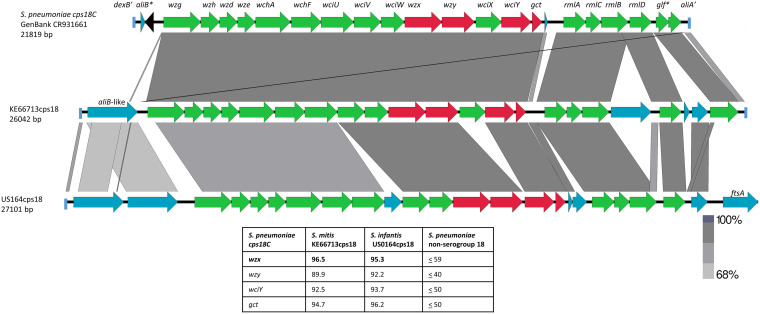
Alignment of *cps* locus from serotype 18C pneumococcus and nonpneumococcal strains KE66713cps18, from a previous study (9), and US164cps18, both recovered from adult OP specimens. The pneumococcal sequence between the 3′ end of *dexB* (*dexB*′) and the 5′ end of *aliA* (*aliA’*) is aligned with corresponding sequences from the nonpneumococcal strains. The *cps* operon genes indicated in green are relatively highly conserved with counterparts from *cps* operons other than from serogroup 18, while the gene alleles in red depict dissimilarity to all other known pneumococcal *cps* genes. Genes indicated in blue are not involved in capsule biosynthesis. Remnants of transposase genes are indicated in black.

Despite PCR assays positive for serogroup 18 *wzy* alleles, we were unable to detect positive Quellung or immunodiffusion results for US164cps18 when employing serogroup 18 pooled or individual pneumococcal serotyping factors. Serogroup 18 (18A, 18B, 18C, and 18F) *cps* loci are representative of the cluster 2 group (40 serotypes that include rhamnose in their capsules) and include the above-described *cps23A* and *cps2* loci, all of which contain rhamnose biosynthetic and transferase genes (*rmlA-D* and *wchF*, respectively, shown in Fig. 3, 4B, and 6).

### Core genome phylogeny of recent PCR serotype-positive nonpneumococcal strains.

**FIG 7:**
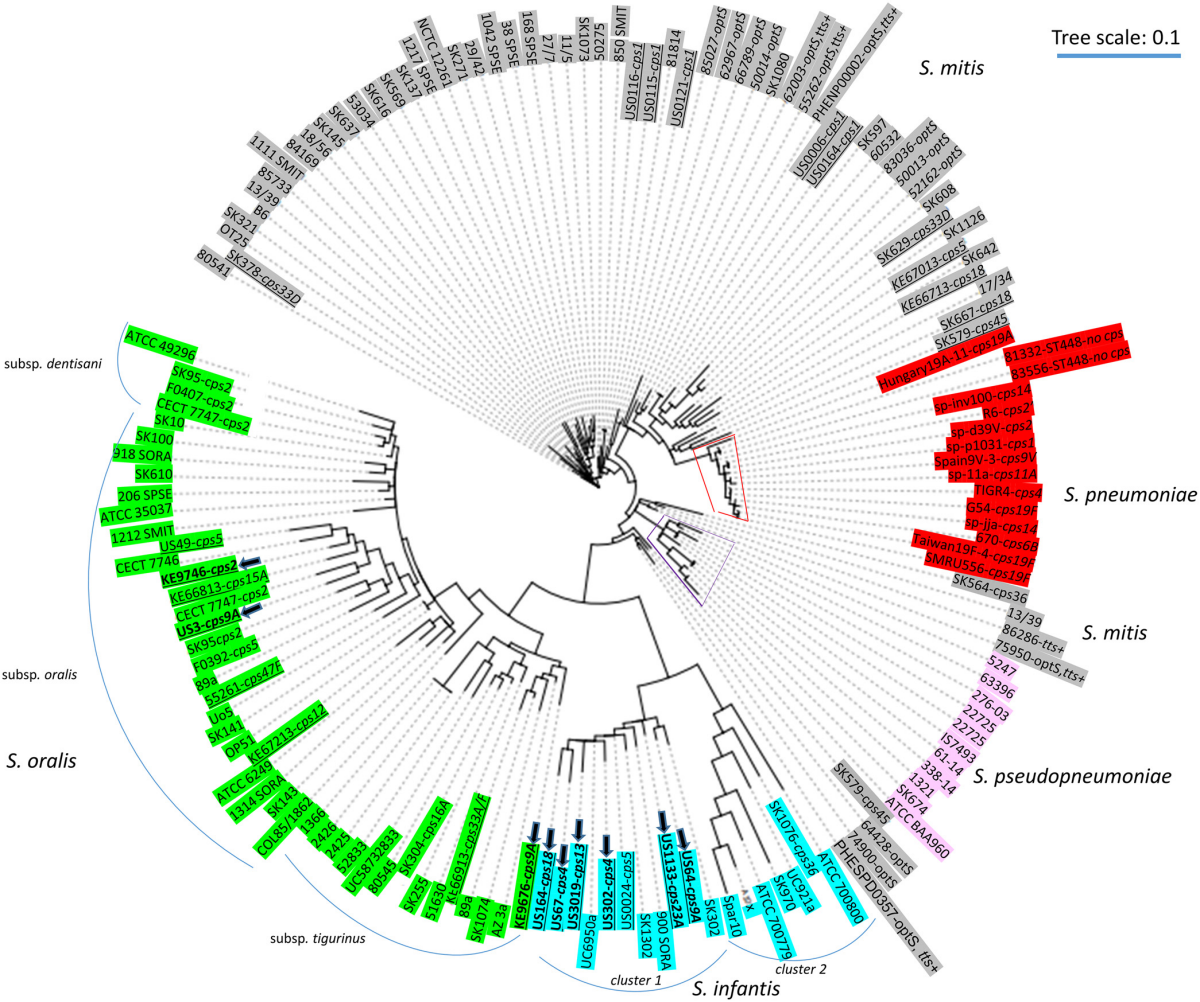
Maximum parsimony core genomic tree based upon kSNP3.0 analysis (33) of genomes accounting for 9 nonpneumococcal carriage isolates carrying homologs of pneumococcal *cps* loci that are the focus of this study are indicated with arrows. Strains from previous studies that carry *cps* loci highly similar to pneumococcal counterparts (9–11) are underlined. All strains are described in Table S1. The k-mer size employed was 19. The scale is based on 322 core single-nucleotide polymorphisms (SNPs). The number of core SNPs was 258. The number of noncore SNPs was 2,043,461. The number of SNPs in at least 0.5 genomes was 77,922. Clusters indicative of S. oralis subspecies and *S. infantis* clusters 1 and 2 are indicated by virtue of described clustering and subspecies designations (28). The species S. pneumoniae and *S. pseudopneumoniae* are indicated in red and purple highlights, respectively. S. mitis, *S. infantis*, and S. oralis are indicated in gray, blue, and green highlights, respectively.

The 9 PCR serotype-positive nonpneumococcal strains described in this work were subjected to genomic sequencing followed by phylogenetic clustering with previously described strains (Fig. 7, Table 2), including nonpneumococcal strains described from other studies (Table S1). To extend the included diversity, 24 invasive nonpneumococcal strains submitted to the CDC Streptococcus laboratory were added to the phylogenetic analysis. These include strains of S. mitis, S. oralis, and *S. pseudopneumoniae.*

**TABLE 2:** Nonpneumococcal strains carrying *cps* locus homologs of pneumococcal strains of known serotypes described in the manuscript

Strain	Yr and country of origin^*a*^	Species/phylogenetic cluster^*b*^	Source of isolate	Capsular serology employing pneumococcal typing sera	Seroreactivity of rabbit antiserum prepared against strain
US302cps4	2015–2016 United States	*S. infantis*/1	Adult OP	Serotype 4	Not tested
US67cps4	2015–2016 United States	*S. infantis*/1	Adult OP	Serotype 4	Specific quelling and killing of serotype 4 pneumococci and of *S. infantis* strain US302cps4
US3cps9A	2015–2016 United States	S. oralis/subsp. *oralis*	Adult OP	Serotype 9A	Not tested
KE9676ps9A	2013 Kenya	S. oralis/subsp. *tigurinis*	Adult OP	Serotype 9A	Not tested
US64cps9A	2015–2016 United States	*S. infantis*/1	Adult OP	Serotype 9A	Specific quelling of pneumococci of all 4 serogroup 9 serotypes; also specific quelling of the 3 *cps9A-*positive nonpneumococcal strains; high killing activity of serotypes 9A and 9V pneumococci and of the 3 nonpneumococcal serotype 9A strains
KE9746ps2	2013 Kenya	S. oralis/subsp. *oralis*	Adult OP	Serotype 2	Not tested
US3019ps13	2019 United States	*S. infantis*/1	Adult NP	Serotype 13	Not tested
US1133ps23A	2015–2016 United States	*S. infantis*/1	Adult OP	Serogroup 23	Specific quelling activity against pneumococci of serogroup 23 serotypes; highest quelling against serotype 23A, followed by 23B and more weakly against 23F; OPK activity
US164cps18	2015–2016 United States	*S. infantis*/1	Adult OP	No reactivity	Not tested

a Isolates recovered during 2015 to 2016 were retrospectively collected from stored specimens from a previously described U.S. adult pneumococcal carriage survey (10, 15). Isolates recovered during 2013 were retrospectively collected from stored specimens from a previously described Kenya pneumococcal carriage survey (14). The isolate recovered during 2019 was originally mistaken for a pneumococcal carriage isolate during a pneumococcal outbreak and was sent to the CDC Streptococcus laboratory for characterization.

b Assignments are based on core genomic phylogeny as shown in Fig. 7.

10.1128/mBio.01037-21.2 TABLE S1 Genome assembly or short read archive accession numbers for strains included within phylogenetic cluster (Fig. 7). To potentially increase the depiction of S. mitis and S. oralis strain diversity, strains were included from the CDC Streptococcus laboratory and reference 30 that were known to have one or more unusual features that included isolation from invasive infections, optochin sensitivity, the presence of the serotype 37-conferring *tts* gene, and the lack of the highly conserved *cps* operon gene *wzg.* The BioProject number for SAMN files from this work as well as references 9 and 10 is PRJNA480039. Species were assigned according to the phylogenetic clustering in Fig. 7. Strains from this and other studies carrying *cps* loci with high similarity to pneumococcal counterparts are indicated. Download Table S1, DOCX file, 0.04 MB. Copyright © 2021 Gertz et al. 2021 Gertz et al. https://creativecommons.org/licenses/by/4.0/ This content is distributed under the terms of the Creative Commons Attribution 4.0 International license.

Six of the 9 PCR serotype-positive strains from this study, including US67cps4, US302cps4, US64cps9A, US164cps18, US1133cps23A, and US3019cps13, formed a cluster that included 4 previously described cluster 1 strains of *S. infantis* (28) and a previously described strain (US24) of serotype 5 *S. infantis* (9). Three strains of S. oralis were apparent, including KE9746cps2 and US3cps9A, that clustered with strains categorized as S. oralis subsp. *oralis* (28). This cluster included 7 additional strains previously identified to carry homologs of S. pneumoniae *cps2*, *cps5*, *cps15A*, *cps47F*, and *cps12* loci (9, 11). Strain KE9676cps9A clustered with previously identified S. oralis subsp. *tigurinus* strains and included strains carrying homologs of pneumococcal *cps16A* (11) and *cps33F* (9).

Figure 7 depicts much of the known diversity of nonpneumococcal strains that carry close homologs of pneumococcal *cps* loci and includes strains that have been previously characterized at genomic and serologic levels (11, 28). Importantly, Fig. 7 depicts 22 nonpneumococcal isolates that we have retrospectively recovered from recent pneumococcal carriage surveys (9, 10, 13–15) that represent 10 different serotypes or serogroups. These include 5 strains within the S. oralis subsp. *oralis* cluster (28) carrying representatives of *cps* homology clusters 1, 4, 6, and 8 (3), including *cps5*, *cps15A*, *cps9A*, *cps12*, and *cps33A-F* recovered in U.S. or Kenya pneumococcal carriage surveys. Five distinct *cps2*^+^ strains (described in references 11 and 12 in addition to KE9746cps2 of this study) shown to express serotype 2 are shown within two of the three S. oralis subspecies shown.

Cluster 1 *S. infantis* (28) also represents broad *cps* diversity with 7 distinct strains carrying homologs of *cps18*, *cps4*, *cps13*, *cps5*, *cps23A*, and *cps9A* of *cps* homology clusters 1, 2, 4, and 6 (3). The presence of two genetically distinct *cps4*^+^ strains within this species is consistent with previous observations that individual *cps* loci are representative of much greater strain diversity within nonpneumococcal species than within pneumococci (9–11, 13).

Evident in this phylogram (Fig. 7) are numerous, diverse strains that are described here and elsewhere (28) as Streptococcus mitis simply by virtue of their close relatedness to, as well as genetic separation from, the two phylogenetically distinct branches that define S. pneumoniae and *S. pseudopneumoniae.* These 3 highly related entities are well demarcated phylogenetically from S. oralis and *S. infantis.* Seven independent S. mitis strains from recent carriage surveys that carry homologs of pneumococcal *cps* operons are depicted, including 5 independent serotype 1 isolates recovered within the United States (10) as well as *cps5*^+^ and *cps18*^+^ strains recovered in Kenya (9). Several additional S. mitis strains with pneumococcal serologic overlap shown in Fig. 7 and Table S1 have been described by Sørensen and colleagues (11). Strains designated S. mitis also included several invasive isolates erroneously submitted to active bacterial core surveillance (ABCs) as pneumococci (unpublished data), some of which carry the *tts* gene, known to confer the Wzy-independent serotype 37 (29), and two similar *tts*^+^ clinical isolates recovered in the United Kingdom (30). It is interesting that the majority of these invasive S. mitis strains also shared the typical pneumococcal phenotype of optochin sensitivity (Table S1).

## DISCUSSION

Here, we build upon data derived from recent pneumococcal carriage surveys confirming that many pneumococcal capsular serotypes are widely dispersed among related commensal species residing in the URT (9–11, 13). Our knowledge of this ecological niche is extremely limited, and here we modestly expand the known shared capsular serology and strain diversity within the subset of the Mitis group that consists of the five species S. pneumoniae, *S. pseudopneumoniae*, S. mitis, S. oralis, and *S. infantis*. This work gives a glimpse of the vast genomic diversity within the individual species of S. mitis, S. oralis, and *S. infantis*, among which expression of pneumococcal capsular serotypes is broadly dispersed. We note that the strains assigned here as S. mitis appear to lack a single major branch point that segregates them as a single species in our phylogenetic analysis.

It is relevant here to provide a summary of recent surveillance-based findings pertaining to the capsular serologic overlap found between pneumococci and close relative species. From relatively limited specimen testing in the United States, we have recovered isolates of commensal Mitis group species carrying homologs of pneumococcal *cps* loci (9 and 10 and this work) that predict vaccine serotypes or serogroups (1, 4, 5, 9V/9A, 18C/18A/18B/18F) and nonvaccine types (23A, 13). Extensive testing of URT specimens from individuals in the United States in the post-PCV era has demonstrated that pneumococcal carriage of PCV serotypes 1, 4, 5, and 9A/9V is rare in both young children and individuals 65 years or older (14, 15, 28, 29). While nonvaccine serotype 13 pneumococci have not been observed in these pneumococcal carriage studies, strains of the nonvaccine serotype 23A have been increasingly prevalent in pediatric carriage (31, 32) and among the top carried strains in older adults (10, 15), although carriage of any pneumococci is uncommon in older adults in the United States. All 5 of these vaccine serotypes or serogroups are also currently rare causes of IPD within the United States, although there has been resurgence of serotype 4 IPD in western states (33). Within ABCs in the year 2018, there were 8 IPD cases due to the nonvaccine serotype 13 and nonvaccine serotype 23A continued to be a major cause of IPD in the post-PCV vaccine era with 125 case isolates (34).

Studies in Kenya that have combined PCR serotype deduction and pneumococcal strain isolation together with limited nonpneumococcal strain isolation have been very informative. From relatively limited URT specimen testing in HIV-positive and HIV-negative adults, we have recovered isolates of commensal species carrying close homologs of pneumococcal *cps* loci (9 and 13 and this work) that predict vaccine serotypes or serogroups (types 2, 5, 9V/9A, 12F/12A/12B, 18C/18A/18B/18F, and 33F/33A) or nonvaccine types (15A/15F). Within certain adult high pneumococcal carriage populations in Kenya, so-called pneumococcal serotype-specific PCR signals from oropharyngeal specimens have been observed to be manyfold higher for numerous serotypes from nonpneumococcal strains than from pneumococcal strains (13). For example, 30 to 40% of adult URT specimens from both HIV-positive (*n* = 118) and HIV-negative (*n* = 40) individuals in Kenya collected during 2009 were PCR2^+^ and PCR5^+^; however, comprehensive attempts to recover serotype 2 or 5 pneumococcal isolates from these specimens as well as from additional specimens collected from adults and young children during 2009 and 2013 have yielded only a few serotype 5 pneumococcal isolates and no serotype 2 pneumococcal isolates (13, 14). In accord with the abundance of their high serotype-specific PCR signal, culture-based screening of a limited number of specimens from this same area (9 and this work) has resulted in recovery of serotype 2 and serotype 5 nonpneumococcal strains (S. oralis strain KE9746cps2 described here and previously described S. mitis strain KE67013cps5 [9]). Although only one specimen collected from 158 adults in this previous study was PCR9A/9V^+^ and several serotype 9V isolates have been recovered from individuals in this area, a serotype 9A S. oralis strain (KE9676cps9A described here) has subsequently been recovered from an adult residing in this region during 2013.

The abundance of these capsular serotypes within nonpneumococcal species appears to coincide with great genomic diversity compared to pneumococci of the same or related serotypes. The known clonal diversity of serotypes 1, 4, 5, and 9A/9V is relatively limited within pneumococci (35, 36) (https://pubmlst.org/). In contrast, the nonpneumococcal isolates of the same serotypes described to date most often are distantly genetically related, with serotypes 5 and 9A even expressed from multiple nonpneumococcal species (this study for serotype 9A; reference 9 describes serotype 5 strains of S. mitis, S. oralis, and *S. infantis*). In a prior study, each of 4 randomly selected 230-bp serotype 2-specific amplicons from individual pneumococcus-negative specimens had a unique sequence that shared 98 to 99% sequence identity to each other and 97 to 98% identity to the corresponding pneumococcal reference *cps2* sequence, suggestive of broad strain diversity (13). Nonpneumococcal serotype 5-specific amplicons from that study revealed even more striking abundance (∼40% of adult specimens) and diversity, with 6 unique 295-bp alleles sequenced that shared 96 to 99% sequence identity to the corresponding highly conserved pneumococcal serotype 5 *cps5* sequence.

There are many questions pertaining to what impact, if any, that the extensive interspecies capsular serotype overlap in the URT has on pneumococcal biology and carriage distributions. Studies to date have only scratched the surface of the extent of this overlap. In the carriage studies discussed (9, 10, 13–15), available URT specimens were screened comprehensively for pneumococcal isolates, while nonpneumococcal isolate screening has only been performed in limited URT specimens. Currently, this area of study lacks nonpneumococcal strain carriage data from younger individuals that more commonly carry pneumococcal strains. Pneumococci of serotypes 1, 2, and 5 were among the most deadly pathogens in the early 1900s (7) yet are currently rare causes of disease in the United States. PCR2^+^ signals have been observed within recent adult carriage specimens in the United States (data not shown); however, at the CDC we have not recovered serotype 2 pneumococci from specimens of any kind during the past 25 years (unpublished data). In view of the absence of serotypes 1 and 5 pneumococci in U.S. carriage specimens from all ages (10, 15, 31, 32), rarity in disease even before PCV implementation (35), and relatively frequent occurrence within nonpneumococcal species, it is tempting to speculate that immune responses to these commensal strains expressing these capsules serve to limit carriage distributions of their pneumococcal counterparts. At this point, such observations are only speculative and are subjective. For example, serotype 23A pneumococci were increasingly carried by children in the post-PCV era (32, 33), and serotype 23A strains of pneumococci were found within adult carriage specimens (10, 15). The nonpneumococcal strain from adult carriage described in this study that was cross-reactive with serotype 23A is likely to be representative of significant carriage of such strains judging from PCR-serotyping assays performed upon pneumococcal-negative carriage specimens. The scenario for serotype 9A carriage among nonpneumococcal strains in the United States is different from that seen for serotypes 1 and 5 in that the nearly identical PCV serotype 9V was commonly carried and a common cause of IPD before PCV implementation (35). Currently, however, it is evident that PCR9A/9V^+^ specimens are found fairly frequently among adults in the United States, and our isolation data strongly suggest that these PCR signals correspond in part or in totality to different commensal nonpneumococcal species expressing serotype 9A.

The increasingly evident capsular serologic overlap between pneumococci and commensal species further highlights that the capsule is not a sole virulence determinant that distinguishes pneumococci from closely related species. Most members of S. mitis carry *cps* loci that are organizationally similar to those within S. pneumoniae. Besides capsule, pneumococci express an array of surface and secreted virulence factors that mediate evasion or exploitation of host immune responses, tissue damage, and invasion (37).

Whether commensal nonpneumococcal strains confer any protective immunity to pneumococci of the same serotypes is a question that is relevant for global pneumococcal strain distributions, past and present. It is conceivable that such naturally available commensal strains could serve as live vaccines. Recently, it has been demonstrated that mucosal immunization of mice with previously described S. mitis serotype 5 (9) induces Th17 and serotype-specific IgG/IgA responses against pneumococcal infection (38). It is not clear what impact current pneumococcal vaccines have on nonpneumococcal strains of vaccine serotypes, although a limited analysis suggested that such strains carrying *cps* loci corresponding to PCV serotypes were less common among vaccinated individuals (10). Key to understanding the underlying seroepidemiology of the Mitis group will be systematic surveillance for commensal strains expressing recognized pneumococcal serotypes in different age groups and populations and an analysis of their carriage-mediated effects upon individual and population immunity to pneumococcal carriage.

## MATERIALS AND METHODS

### Strains and genomic sequencing.

All 9 isolates described in this study, carrying homologs of pneumococcal *cps* operons, were recovered from PCR serotype-positive URT specimens that were negative for the pneumococcal *lytA* qPCR assay (9, 10). These strains were classified as nonpneumococcal based upon optochin resistance, bile insolubility, and the absence of the *piaA* gene, which is highly conserved and ubiquitous among encapsulated pneumococcal strains (39). Eight of the nine isolates were recovered from OP specimens and 1 from an NP specimen. These 9 strains and 28 additional isolates recovered through ABC’s IPD surveillance (26 invasive nonpneumococcal strains sent to verify as nonpneumococcal, two nontypeable pneumococci) were subjected to genomic sequencing on the Illumina MiSeq platform as previously described (33, 34, 36). Accession numbers for the 9 isolates that are the focus of this study, and 28 additional strains employed for phylogenetic analysis are provided in Table S1 in the supplemental material.

### U.S. nonpneumococcal carriage strains.

Four hundred sixty paired respiratory OP and NP swabs from a pneumococcal carriage study in the United States carried out during 2015 to 2016 in healthy adults aged 65 years and older were assessed for pneumococcal carriage using culture-based methods (10, 15) and 450 of these individuals were culture negative for pneumococci. A limited *lytA* qPCR-negative subset of the corresponding 450 paired specimens was assessed for nonpneumococcal strains positive for certain PCR serotypes detected by sequential cPCR and qPCR assays (19–21) (updates at https://www.cdc.gov/streplab/pneumococcus/resources.html). From this U.S. carriage study, nonpneumococcal strains US67cps4, US302cps4, US64cps9A, US3cps9A, US164cps18, and US1133cps23A were obtained from stored OP swab specimens and isolated as previously described (9, 10). Strain US3019cps13 was collected from an adult NP carriage specimen during an investigation of an IPD outbreak in a U.S. prison during 2019 (our unpublished data).

### Kenya nonpneumococcal carriage strains.

Nonpneumococcal strain KE9746cps2 and KE9676cps9A were retrospectively recovered from stored adult OP specimens (2,028 of 2,590 [78.3%] individuals were HIV positive) collected during 2013 from a carriage study in Kenya (14). We lack information linking these isolates with HIV status.

### *cps* operon alignments.

Reference pneumococcal *cps* operons used in figures are described in depth in references 3 and 5. Each of these reference loci lay between the *dexB* 3′ end and the *aliA* 5′ ends. Corresponding genomic segments were extracted from genomic sequences of the 9 nonpneumococcal strains that were the study’s focus and subjected to Prokka to identify and annotate open reading frames (40). The annotated sequences were analyzed by BLAST, and the *cps* regions were aligned into figures using EasyFig 2.2.3 (41).

### Species assignments

Strains were assigned species through clustering with previously speciated strains (9, 28) employing core genomic kSNP3.0 (42) analysis to generate a single-nucleotide polymorphism matrix and a parsimony phylogenetic tree that represents a consensus tree and node support based on an extended majority rule consensus of the equally most parsimonious trees from a sample of 100 trees. A phylogenic tree was generated employing the iTOL (43) program (https://itol.embl.de/).

### Institutional approvals.

Centers for Disease Control and Prevention (CDC) and local institutional review boards approved the studies (CDC protocol number 6725), and it was approved by ethics committees as described previously (9, 14, 15).

### Preparation of streptococcal antisera.

This protocol (number 3028GERRABC) was approved by the Institutional Animal Care and Use Committee (IACUC). Antisera against formalin-fixed strains US67cps4, US64cps9A, and US1133cps23A were prepared exactly as described for CDC capsular typing antisera prepared against pneumococcal strains (44).

### Serology.

Latex agglutination and the Quellung reaction employing CDC rabbit polyclonal typing antiserum were used as described previously (44). Double immunodiffusion assays employing pneumococcal typing sera and antisera prepared against commensal streptococci were carried out as previously described (9, 10) using Pierce standard Ouchterlony agarose gel plates (Pierce number 31111; Thermofisher Scientific).

### OPK assays.

OPK assays were performed employing HL-60 cells and complement source (baby rabbit serum; Pel-Freez, Brown Deer, WI) as outlined previously (45). Bacterial strains were grown overnight in Todd-Hewitt broth supplemented with 0.5% yeast extract. A 500-μl aliquot was incubated in 25 ml of the same growth medium and grown to an optical density at 650 nm of 0.4. Glycerol was added to each culture to a final concentration of 20% before dispensing 1-ml aliquots in cryovials and flash freezing in an ethanol-dry ice bath. Frozen cultures were kept at ≤–70°C for further use. Viable counts of aliquots were performed to determine the concentration of pneumococci before and after the freezing protocol, and 1,000 CFU/20 μl was used in each OPK assay. For each nonpneumococcal strain (US64cps9A and US67cps4), serum samples were obtained from 2 to 3 rabbits inoculated over a period of 6 weeks with the formalin-fixed vaccine. Pooled samples from 2 to 3 animals were used for OPK experiments following verification of capsule expression by immunodiffusion and Quellung reaction against the reference serotype 4 and serogroup 9 S. pneumoniae strains.

Geometric mean titer (GMT) values with >50% killing compared with the growth in the complement control wells were generated across 4 to 8 assay runs. For serotype 4 pneumococcal typing serum, antiserum against US67cps4, pooled pneumococcal serogroup 9 antiserum, and antiserum against US64cps9A, 2-fold dilutions (1/8 through 1/1,024) and 3-fold dilutions (1/400 through 1/874,8000) were employed. An additional 2-fold series from 1:400 (1:400 through 1:51,200) of pneumococcal type 4 antiserum was also used. Given the lack of response with types 9L and 9N, we additionally assayed high concentrations (from 1:1 through 1:128) for serogroup 9 strains.

### Uronic acid assay.

Glucuronic acid within serotypes 2 and 9A was assayed by a method that exploits the appearance of a chromogen when uronic acid is heated to 100°C in concentrated sulfuric acid-tetraborate followed by the addition of meta-hydroxydiphenyl (22), as used for bacterial suspensions (23, 24). Strains were grown on trypticase soy agar containing 5% sheep’s blood plates overnight at 37°C, 5% CO_2_, which was used to inoculate Todd Hewitt broth plus neopeptone (THBN) to mid-log phase and then held overnight at 4°C. The next day, 500 μl was transferred to 30 ml THBN at 37°C, 5% CO_2_ and incubated to the end of log phase. Dilution was used to standardize the *A*_660_ readings to 0.5. Aliquots of 10 ml were then centrifuged at 8,000 × *g* to obtain pellets. Pellets were washed 3× in 1 ml 1× phosphate-buffered saline (PBS) at 4°C at 8,000 × *g*. Pellets were resuspended in 1 ml of 150 mM Tris-HCl, pH 7, and 1 mM MgSO_4_ and centrifuged again at 4°C at 8,000 × *g*. The supernatant was removed, and the pellets were resuspended in 500 μl of 150 mM Tris-HCl, pH 7, and 1 mM MgSO_4_, and 200 μl was transferred to a new tube; 1.2 ml of 12.5 mM sodium tetraborate in concentrated sulfuric acid was added, and the tubes were mixed and then heated in a hot bath at 100°C for 5 min and transferred to an ice bath. A blank of 200 μl 150 mM Tris-HCl, pH 7, 1 mM MgSO_4_, and 1.2 ml 12.5 mM sodium tetraborate was prepared and heated to 100°C for 5 min. Twenty microliters of 0.15% m-hydrobiphenyl in 0.5% NaOH was added to each sample, with 20 μl of 0.5% NaOH substituted in the blank. The samples were immediately transferred to borosilicate cuvettes and read at 520 nm. Standards of glucuronic acid were prepared in water, including 1 mg/ml, 0.1 mg/ml, 0.02 mg/ml, 0.01 mg/ml, and 0.005 mg/ml, and, as with the samples, 200 μl of each preparation was added to 12.5 mM sodium tetraborate in concentrated sulfuric acid, mixed well, heated to 100°C, transferred to an ice bath, had 20 μl 0.15% m-hydrobiphenyl in 0.5% NaOH added, and were transferred to borosilicate cuvettes for readings at 520 nm.

### Data availability.

The BioProject number for SAMN files from this work as well as references 7 and 8 (all numbers are provided in Table S1) is PRJNA480039.
