# Supplementary figures and images for: Nonpneumococcal Strains Recently Recovered from Carriage Specimens and Expressing Capsular Serotypes Highly Related or Identical to Pneumococcal Serotypes 2, 4, 9A, 13, and 23A
Source: mBio. 2021 May 18;12(3):e01037-21. doi: 10.1128/mBio.01037-21 (PMC8262907; doi:10.1128/mBio.01037-21)

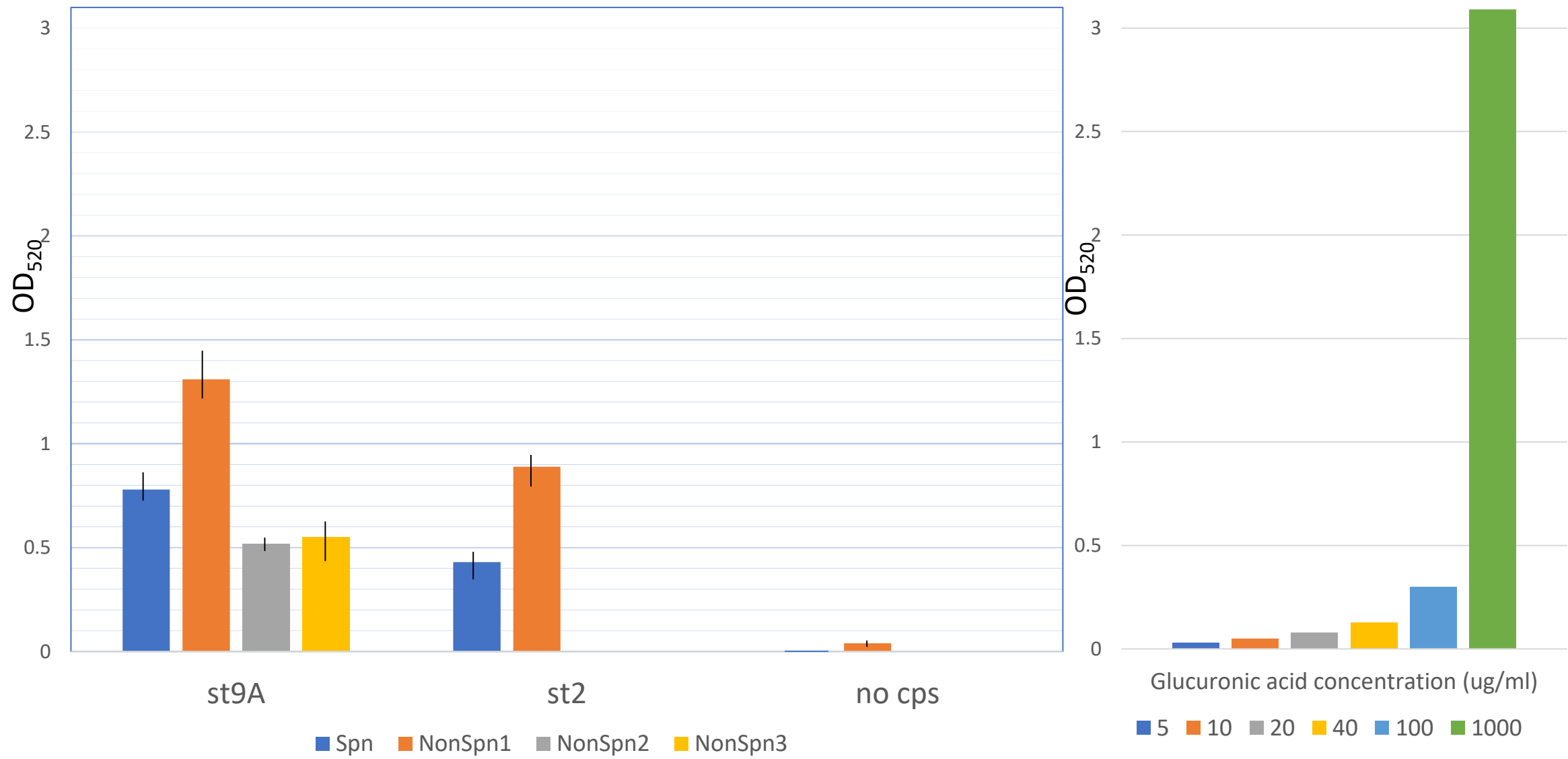

supplementary Figure 1

Supplement: FIG S1 [file mbio.01037-21-sf001.pdf]
