# Supplementary material for: Nonpneumococcal Strains Recently Recovered from Carriage Specimens and Expressing Capsular Serotypes Highly Related or Identical to Pneumococcal Serotypes 2, 4, 9A, 13, and 23A
Source: mBio. 2021 May 18;12(3):e01037-21. doi: 10.1128/mBio.01037-21 (PMC8262907; doi:10.1128/mBio.01037-21)
Supplement: TABLE S1 [file mbio.01037-21-st001.docx]

STable 1.  Genome assembly or short read archive accession numbers for strains included within phylogenetic depiction (Figure 7).

| Strain^A^ | Species (features) ^A^ | Reference | NCBI genome accession reference for assemblies, or short read archive reference (SAMN)^B^ |
| --- | --- | --- | --- |
| US302cps4 | *S. infantis* (*cps4,* carriage) | this work | SAMN16913259 |
| US67cps4 | *S. infantis* (*cps4,* carriage) | this work | SAMN16913257 |
| US3cps9A | *S. oralis* (*cps9A*, carriage) | this work | SAMN16913255 |
| KE9676cps9A | *S. oralis* (*cps9,* carriage) | this work | SAMN16913253 |
| US64cps9A | *S. infantis* (*cps9*, carriage) | this work | SAMN16913256 |
| KE9746cps2 | *S. oralis* (*cps2*, carriage) | this work | SAMN16913254 |
| US3019cps13 | *S. infantis* (*cps13*, carriage) | this work | SAMN16913252 |
| US1133cps23A | *S. infantis* (*cps23A* carriage) | this work | SAMN16913260 |
| US164cps18 | *S. infantis* (*cps18*, carriage) | this work | SAMN16913258 |
| US116 | *S. mitis* (*cps1*, carriage) | 8 | SAMN09624062 |
| US115 | *S. mitis* (*cps1*, carriage) | 8 | SAMN09624061 |
| US6 | *S. mitis* (*cps1*, carriage) | 8 | SAMN09624059 |
| US164 | *S. mitis* (*cps1*, carriage) | 8 | SAMN09624060 |
| US121 | *S. mitis* (*cps1*, carriage) | 8 | SAMN09624063 |
| KE67013 | *S. mitis* (*cps5,* carriage) | 7 | SAMN09874921 |
| US0049 | *S. oralis* (*cps5,* carriage) | 7 | SAMN09874924 |
| US969j1 | *S. infantis* (*cps5,* carriage) | 7 | SAMN09874925 |
| US0024H | *S. infantis* (*cps5,* carriage) | 7 | SAMN09874926 |
| KE66913 | *S. oralis* (*cps33F,* carriage) | 7 | SAMN09874920 |
| KE66713 | *S. mitis* (*cps18,* carriage) | 7 | SAMN09874921 |
| KE67213 | *S. oralis* (*cps12,* carriage) | 7 | SAMN09874922 |
| KE66813 | *S. oralis* (*cps15A,* carriage) | 7 | SAMN09874923 |
| 55261 | *S. oralis* (*cps47F,* invasive) | this work | SAMN16913234 |
| 50014 | *S. mitis* (invasive, optochin-sensitive, *wzg-negative*) | this work | SAMN16913229 |
| 52162 | *S. mitis (invasive, optochin-sensitive, wzg-negative)* | this work | SAMN16913231 |
| 53034 | *S. mitis* (invasive) | this work | SAMN16913233 |
| 62967 | *S. mitis (invasive, optochin-sensitive, wzg-negative)* | this work | SAMN16913238 |
| 64428 | *S. mitis (invasive, optochin-sensitive, wzg-negative)* | this work | SAMN16913240 |
| 66789 | *S. mitis (invasive, optochin-sensitive, wzg-negative)* | this work | SAMN16913241 |
| 74900 | *S. mitis (invasive, optochin-sensitive, wzg-negative)* | this work | SAMN16913242 |
| 75950 | *S. mitis (invasive, optochin-sensitive, wzg-negative)* | this work | SAMN16913243 |
| 80541 | *S. mitis* (invasive, optochin-sensitive) | this work | SAMN16913244 |
| 81814 | *S. mitis* (invasive) | this work | SAMN16913246 |
| 83036 | *S. mitis (invasive, optochin-sensitive, wzg-negative)* | this work | SAMN16913247 |
| 85733 | *S. mitis* (invasive) | this work | SAMN16913250 |
| 84619 | *S. mitis* (invasive) | this work | SAMN16913248 |
| 85027 | *S. mitis (invasive, optochin-sensitive, wzg-negative)* | this work | SAMN16913249 |
| 55262 | *S. mitis* (*tts*, optochin-sensitive, *wzg-*negative, invasive) | this work | SAMN16913235 |
| 62003 | *S. mitis* (*tts*, optochin-sensitive, *wzg-*negative, invasive) | this work | SAMN16913237 |
| 86286 | *S. mitis* (*tts*, *wzg-*negative, invasive) | this work | SAMN16913251 |
| 50013 | *S. mitis (invasive, optochin-sensitive, wzg-negative)* | this work | SAMN16913228 |
| 60532 | *S. mitis (invasive, optochin-sensitive, wzg-negative)* | this work | SAMN16913236 |
| 80545 | *S. oralis* (invasive) | this work | SAMN16913245 |
| 51630 | *S. oralis* (invasive) | this work | SAMN16913230 |
| 52833 | *S. oralis* (invasive) | this work | SAMN16913232 |
| 63396 | *S. pseudopneumoniae* (invasive) | this work | SAMN16913239 |
| sp81332 | *S.pneumoniae* (ST448,acapsular) | this work | SAMN11121649 |
| sp83556 | *S.pneumoniae* (ST448,acapsular) | this work | SAMN11122743 |
| sp-inv200 | *S. pneumoniae* (*cps14*) | 20 | FQ312029 |
| Spain9V-3 | *S. pneumoniae* (*cps9V*) | 20 | ABGE00000000 |
| sp-d39v | *S. pneumoniae* (*cps2*) | 20 | CP027540 |
| sp-11a | *S. pneumoniae* (*cps11A*) | 20 | CP018838 |
| SMRU556 | *S. pneumoniae* (*cps19F*) | 20 | CP003357 |
| G54 | *S. pneumoniae* (*cps19F*) | 20 | CP001015 |
| sp-p1031 | *S. pneumoniae* (*cps1*) | 20 | CP000920 |
| sp-jja | *S. pneumoniae* (*cps14*) | 20 | CP000919 |
| 670-6B | *S. pneumoniae* (*cps6B*) | 20 | CP002176 |
| Hungary19A-6 | *S. pneumoniae* (*cps19A*) | 20 | CP000936 |
| Taiwan19F-4 | *S. pneumoniae* (*cps19F*) | 20 | CP000921 |
| Tigr4 | *S. pneumoniae* (*cps4*) | 20 | AE005672 |
| R6 | *S. pneumoniae* (*cps2-*defective) | 20 | AE007317 |
| SK667 | *S. mitis* (*cps18*) | 9 | JPFY00000000 |
| SK564 | *S. mitis* (*cps36*) | 9 | AEDU00000000 |
| SK578 | *S. mitis* (*cps33D*) | 9 | JPFY00000000 |
| SK629 | *S. mitis* (*cps33D*) | 9 | JPFU00000000 |
| SK579 | *S. mitis* (*cps45*) | 9 | AJJL00000000 |
| SK575 | *S. mitis* (*cps45*) | 9 | ICU00000000 |
| SK616 | *S. mitis* (*cps45*) | 9 | AICR00000000 |
| PHENP00002 | *S. mitis* (*tts,* optochin sensitive, *wzg-*negative, invasive) | 22 | SAMEA103985790 |
| PHESPD0357 | *S. mitis (tts, optochin sensitive, wzg-negative, invasive)* | 22 | SAMEA103985791 |
| SK95 | *S. oralis* (*cps2*) | 9 | AFUB00000000 |
| CECT 7747 | *S. oralis* (*cps2*) | 9 | CAUK00000000 |
| F0407 | *S. oralis* (*cps2*) | 9 | JH378872 |
| F0392 | *S. oralis* (*cps5*) | 9 | CP034442 |
| SK304 | *S. oralis* (*cps16A*) | 9 | ALJN00000000 |
| SK1076 | *S. infantis* (*cps36*) | 9 | AFNN00000000 |
| SK1080 | *S. mitis* | 20 | AFQV00000000 |
| SK1073 | *S. mitis* | 20 | AFQT00000000 |
| NCTC 12261 | *S. mitis* | 20 | UHFS00000000 |
| SK597 | *S. mitis* | 20 | AEDV00000000 |
| SK321 | *S. mitis* | 20 | AEDT00000000 |
| B6 | *S. mitis* | 20 | FN568063 |
| 27/7 | *S. mitis* | 20 | AYRS01000000 |
| 13/39 | *S. mitis* | 20 | AQTU01000000 |
| SK569 | *S. mitis* | 20 | AFUF01000000 |
| 1042 SPSE | *S. mitis* | 20 | JWFA00000000 |
| 1111 SMIT | *S. mitis* | 20 | JWCV00000000 |
| 11/5 | *S. mitis* | 20 | AQTT00000000 |
| SK642 | *S. mitis* | 20 | JPFW00000000 |
| 1217 SPSE | *S. mitis* | 20 | JVWP01000000 |
| 13/39 | *S. mitis* | 20 | AQTU00000000 |
| 168 SPSE | *s. mitis* | 20 | JVRN00000000 |
| 17/34 | *S. mitis* | 20 | ASZZ00000000 |
| 18/56 | *S. mitis* | 20 | ATAA00000000 |
| 29/42 | *S. mitis* | 20 | ATAB00000000 |
| 38 SPSE | *S. mitis* | 20 | JVJJ00000000 |
| 50275 | *S. mitis* | 20 | NCVF00000000 |
| OT25 | *s. mitis* | 20 | JYGP00000000 |
| 850 SMIT | *S. mitis* | 20 | JUQO00000000 |
| SK1126 | *S. mitis* | 20 | JPFT00000000 |
| SK145 | *S. mitis* | 20 | JYGS00000000 |
| SK137 | *S. mitis* | 20 | JPFS00000000 |
| SK271 | *S. mitis* | 20 | JPGW00000000 |
| SK608 | *S. mitis* | 20 | JPFZ00000000 |
| SK637 | *S. mitis* | 20 | CP028415 |
| ATCC 6249 | *S. oralis* | 20 | AEEN01000000 |
| 1212 SMIT | *S. oralis* | 20 | JVYP00000000 |
| 1314 SORA | *S. oralis* | 20 | JVUD00000000 |
| 1366 | *S. oralis* | 20 | AORX00000000 |
| COL85/1862 | *S. oralis* | 20 | JYGM00000000 |
| 206 SPSE | *S. oralis* | 20 | JVPZ00000000 |
| ATCC 35037 | *S. oralis* | 20 | GG749277 |
| ATCC 49296 | *S. oralis* | 20 | GL622183 |
| UC5873 | *S. oralis* | 20 | JYGU00000000 |
| 727 SORA | *S. oralis* | 20 | JUVM00000000 |
| 734 SORA | *S. oralis* | 20 | JUVF00000000 |
| CECT 7746 | *S. oralis* | 20 | CAUJ00000000 |
| 89a | *S. oralis* | 20 | LKPC00000000 |
| 918 SORA | *S. oralis* | 20 | JUNW00000000 |
| OP51 | *S. oralis* | 20 | JYGO00000000 |
| SK100 | *S. oralis* | 20 | AJKP00000000 |
| SK1074 | *S. oralis* | 20 | AICT00000000 |
| SK10 | *S. oralis* | 20 | AJKO00000000 |
| SK141 | *S. oralis* | 20 | JPGA00000000 |
| SK143 | *S. oralis* | 20 | JPGB00000000 |
| SK255 | *S. oralis* | 20 | AFNM00000000 |
| SK610 | *S. oralis* | 20 | AJKQ00000000 |
| Uo5 | *S. oralis* | 20 | FR720602 |
| 2425 | *S. oralis* | 20 | ASWZ00000000 |
| 2426 | *S. oralis* | 20 | ASXA00000000 |
| 274 SPSE | *S. oralis* | 20 | JVNJ00000000 |
| AZ 3a | *S. oralis* | 20 | AORU00000000 |
| SK674 | *S. pseudopneumoniae* | 20 | AJKE00000000 |
| 276-03 | *S. pseudopneumoniae* | 20 | LJHJ00000000 |
| 61-14 | *S. pseudopneumoniae* | 20 | LJHK00000000 |
| 338-14 | *S. pseudopneumoniae* | 20 | LJHI00000000 |
| 5247 | *S. pseudopneumoniae* | 20 | AYRQ00000000 |
| 22725 | *S. pseudopneumoniae* | 20 | AYRO00000000 |
| 1321 | *S. pseudopneumoniae* | 20 | AYRP00000000 |
| ATCC BAA960 | *S. pseudopneumoniae* | 20 | AICS00000000 |
| IS7493 | *S. pseudopneumoniae* | 20 | CP002925 |
| 22725 | *S. pseudopneumoniae* | 20 | AYRO00000000 |
| UC6950A | *S. infantis* | 20 | JYOV00000000 |
| ATCC 700780 | *S. infantis* | 20 | GL732463 |
| 900 SORA | *S. infantis* | 20 | JUOS00000000 |
| ATCC 700779 | *S. infantis* | 20 | GL732439 |
| SK1302 | *S. infantis* | 20 | AEDY00000000 |
| SK970 | *S. infantis* | 20 | AFUT00000000 |
| Spar10 | *S. infantis* | 20 | ALCH00000000 |
| UC921a | *S. infantis* | 20 | JYGT00000000 |
| x | *S. infantis* | 20 | AFUQ00000000 |

^A^ In order to potentially increase the depiction of *S. mitis* and *S. oralis* strain diversity, strains were included from the CDC *Streptococcus* Lab and reference 30 that were known to have one or more unusual features that included isolation from invasive infections, optochin-sensitivity, the presence of the serotype 37 – conferring *tts* gene, and the lack of the highly conserved *cps* operon gene *wzg.*

^B^ Bioproject number for SAMN files from this work as well as references 7-8 is PRJNA480039. Species were assigned according to the phylogenetic clustering in Figure 7.
